# Incubation Temperature during Fetal Development Influences Morphophysiological Characteristics and Preferred Ambient Temperature of Chicken Hatchlings

**DOI:** 10.1371/journal.pone.0154928

**Published:** 2016-05-16

**Authors:** Viviane de Souza Morita, Vitor Rosa de Almeida, João Batista Matos, Tamiris Iara Vicentini, Henry van den Brand, Isabel Cristina Boleli

**Affiliations:** 1 Department of Animal Morphology and Physiology, Sao Paulo State University, Access road Professor Paulo Donato Castellane, s/n, 14884–900, Jaboticabal, Sao Paulo, Brazil; 2 Adaptation Physiology Group, Department of Animal Sciences, Wageningen University, PO Box 338, 6700 AH, Wageningen, The Netherlands; University of Florida, UNITED STATES

## Abstract

Skin and feather characteristics, which play a critical role in body temperature maintenance, can be affected by incubation circumstances, such as incubation temperature. However, no study to date has assessed the influence of incubation temperature during the fetal stage on morphometric characteristics and vascular development of the skin, feather characteristics, and their relationship to hormone levels and preferred temperature in later life in chickens. Broiler breeder eggs were exposed to low (36°C), control (37.5°C), or high (39°C) temperatures (treatments LT, CK, and HT, respectively) from day 13 of incubation onward, because it is known that the endocrine axes are already established at this time. During this period, eggshell temperature of HT eggs (38.8±0.33°C) was higher than of LT (37.4±0.08°C) and CK eggs (37.8 ±0.15°C). The difference between eggshell and incubator air temperature diminished with the increasing incubation temperature, and was approximately zero for HT. HT hatchlings had higher surface temperature on the head, neck, and back, and thinner and more vascularized skin than did CK and LT hatchlings. No differences were found among treatments for body weight, total feather weight, number and length of barbs, barbule length, and plasma T4 concentration. LT hatchlings showed lower plasma T3 and GH, as well as lower T3/T4 ratio and decreased vascularity in the neck, back, and thigh skin compared to CK hatchlings. On the other hand, HT hatchlings had decreased skin thickness and increased vascularity, and preferred a higher ambient temperature compared to CK and HT hatchlings. In addition, for all treatments, surface temperature on the head was higher than of the other body regions. We conclude that changes in skin thickness and vascularity, as well as changes in thyroid and growth hormone levels, are the result of embryonic strategies to cope with higher or lower than normal incubation temperatures. Additionally exposure to increased temperature during incubation is an environmental factor that can exert early-life influence on ambient temperature preference of broiler hatchlings in later life.

## Introduction

In precocial birds, such as broilers, the quality and survival of the neonate depend on maternal investment in egg characteristics and incubation temperature [1,2,3], as well as on care during the early post-hatching period. Under natural conditions, neonates require the maternal presence to maintain body temperature during the transition from ectothermy to homeothermy. Thermal discomfort may influence the health and well-being of the animal, as well as their growth and survival. The integument plays a key role in homeostasis, as changes in dermal blood flow and surface temperature are involved in adjusting the body temperature [4, 5, 6, 7]. In addition, feathering aids in development of homeothermy by providing a natural insulation layer. Natural feathering alterations occur, as the replacement of down feathers by adult contour feathers is influenced by bird metabolism [8]. Feathering pattern modifications may also occur in response to excessive cold or heat in order to prevent or enhance heat dissipation, respectively [9]. Structural changes in the bird skin related to heat exposure were also reported as response to changes in temperature. Heat-acclimated pigeons demonstrated increased dermis vascularization, thicker epidermis and changes in the intracellular structure compared to non-acclimated pigeons [10].

In commercial broiler production, maternal egg incubation is replaced by artificial incubation, during which temperatures remain constant to ensure high hatchability [1]. However, several studies have shown that a 1–2°C intermittent increase in the usual incubation temperature (37.5–37.8°C) can alter offspring phenotype by improving thermal tolerance throughout adulthood [11, 12, 13, 14, 15, 16, 17]. Additionally, utilization of incubation temperatures 1°C above usual after day 7 of incubation increases the feather follicle density on neonate backs [18]. Besides, recently it was showed that incubation temperature 1°C below usual (37.5°C) during the first three days and 1°C above usual during the last three days of incubation also increased the feather follicle density on the back and thighs of chickens at 22 days of life [19].

Skin and feather alterations occur in parallel with or are preceded by hormonal changes. In mammals, skin characteristics are influenced by thyroid and growth hormones [20]. In birds, it is known that T3 and T4 hormones influence cell growth in feather follicles [21], and it has also been reported that changes in incubation temperature influence plasma thyroid hormone and growth hormone concentrations in neonate chickens [22]. Thus, it can be suggested that incubation temperature indirectly affects feather follicle development via changes in thyroid hormone and growth hormone concentrations.

Although the bird skin responds to changes in incubation temperature, morphophysiological adjustments in the skin related to homeothermy, which might be related to behavioral changes, such as the search for sites with appropriate temperatures, were not reported in the literature. It was verified that turkey hatchlings from eggs incubated at 38.5°C from day 7 of incubation to hatch preferred higher temperatures than hatchlings from eggs incubated at 37.5°C [23]. Additionally, layer chicken hatchlings obtained from eggs incubated at 40°C for 4 h/day from day 14 to day 18 of incubation preferred, at day seven after hatching, an ambient temperature 1°C lower than hatchlings incubated at a constant eggshell temperature of 37.8°C [24]. More recently, it was shown that broiler hatchlings, obtained from eggs incubated at 34°C preferred an ambient temperature 1°C higher than hatchlings obtained from eggs incubated at 37.5°C, but their body weights and number of vocalizations were not influenced [25]. However, these studies did not investigated the occurrence of structural skin changes in response to incubation temperature manipulation.

Despite the vast number of studies on the effects of incubation temperature, no attempts have been made: (1) to assess potential effects of incubation temperature on neonate skin thickness and vascularity as well as on feather morphological characteristics; (2) to associate changes in skin and feather structural characteristics with underlying hormonal rates; and (3) to associate structural and physiological changes with different temperature preferences after hatching.

We hypothesized that continuous exposure to cold or heat during the last days of incubation can induce a cascade of morphophysiological changes, which in turn, would affect hatchling thermal preference. To test this hypothesis, we investigated whether a continuously higher or lower temperature starting at day 13 of incubation affects skin and feather characteristics, body surface and rectal temperatures, plasma thyroid hormone and growth hormone concentrations, and preferred ambient temperature after hatching.

## Materials and Methods

### Ethical standards

All experimental procedures used in this study were approved by the local Ethics Committee for the Use of Animals (CEUA- protocol n° 021086/11; FCAV/UNESP, Jaboticabal, Brazil).

### Incubation

A total of 780 fertile eggs from a 59 weeks old broiler breeder flock (Cobb 500 fast feathering) were obtained from a commercial hatchery (Globoaves, Itirapina, São Paulo, Brazil), weighed, and distributed homogeneously by weight (69.5±2.5g) over six incubators (Premium Ecológica; 130 eggs per incubator. Incubation temperature for all incubators was set at 37.5°C until day 12 of incubation. From day 13 until hatching, the incubation temperature was lowered to 36°C (treatment LT), increased to 39°C (treatment HT), or maintained at 37.5°C (treatment CK), resulting in two incubators per incubation temperature profile. These incubation temperatures corresponded to eggshell temperatures of 37.4±0.08°C, 38.8±0.33°C and 37.8 ±0.15°C, respectively, which were measured every 30 min from d 13 until d 19 of incubation and stored in data loggers connected to a computer. Relative humidity in all incubators was maintained at 60% throughout incubation. Egg turning each 2 h was maintained until day 18 of incubation. At day 18, all eggs were moved to hatching baskets, which were placed in the same incubators. The applied incubation temperatures were used to induce thermal programming. Thermal programming from day 13 of incubation onward was determined based in the knowledge that functional maturation of the hypothalamo-pituitary-thyroid axis is established around 13–14 days of incubation [26]. Because thyroid hormones influence the metabolism, development, and thermoregulatory mechanisms of the chicks in the post-hatch period [26], thermal manipulation during this embryonic development period may be an effective tool to induce morphological and physiological changes in the chicks that improve their heat tolerance during adulthood [27].

### Eggshell temperature, incubation duration and hatchability

At every fifth egg within each incubator, a thermistor (Alutal Type T; São Paulo, Brazil) was attached to the shells at the equatorial region of the eggs, covered with Styrofoam, and attached with regular cellophane tape (3M) for the monitoring of eggshell temperature. Additionally, incubator air temperature was monitored in the central region of the incubator. Eggshell and incubator air temperatures were measured every 30 min from onset of incubation until day 19 of incubation and stored in data loggers connected to a computer for further analysis. Incubator thermostat temperature was measured daily at 8 AM and 5 PM over the same time period as for eggshell and incubator air temperatures.

From day 19 of incubation (456 h of incubation), the incubators were checked every three hours to determine individual incubation time (hour) per chick. Incubation time corresponded to the number of hours between the start of incubation and hatching. After day 22 (528 h of incubation), unhatched eggs were analyzed for fertility or embryo mortality using the classification table of Lourens et al. [28]. After drying (3 hour after hatch), all hatchlings were sexed by examining the feathers as described by the Broiler Management Guide [29]. Because sex-can affect incubation length and erythrocyte values, as shown in a previous study [30], and consequentely can influence the effects of temperature manipulation, only males were analyzed in the present study.

### Body surface and rectal temperatures

Immediately after sexing, male hatchlings were weighed and 24 chicks per treatment (12 chicks per incubator) with average body weight (48.00 ± 1.00 g) were used for blood collection and skin analysis. Infrared thermal images of these 24 chicks per treatment were collected for determination of surface temperature of the head (cranial), neck, back (thoracic-dorsal), breast (chest-ventral) and thigh, using an infrared thermography camera with a precision of ± 0.7°C and spectrum range of 7.5–13 μm (FLIR E40; Stockholm, Sweden). To obtain and analyze thermography images, a methodology utilized in a previous study was adopted [7]. For better visualization of the images and to avoid visual interference from the surrounding area, cardboard was placed behind each chick. The thermal camera was placed approximately 1 m from the bird and the image was obtained from an angle of 90° from the chick surface. These procedures were utilized so that the image would fill the field of view and to minimize errors due to angle distortion, respectively. As proposed in an earlier study [6], an emissivity coefficient (ε) of 0.95 was used. The surface temperature of each of the five regions corresponded to the average temperature measured at several randomly chosen points on the head (13 points), neck (10 points), back (10 points), breast (12 points) and thigh (8 points) according to a method adapted from a study developed previously [7]. Each thermal image was analyzed using software provided by the camera manufacturing company (FLIR Tools, Stockholm, Sweden). Rectal temperature was measured on the same chickens, using a digital thermometer (MedeQCO, Geschwenda, Germany, with an accuracy of ± 0.1°C) inserted through the cloaca for approximately 3 cm. Rectal temperature has been used to estimate the body temperature in chickens [31]. To avoid effects of incubation treatments, all procedures were performed within acrylic climatic chambers (80 x 80 x 80 cm, with automatic temperature control), whose air temperature was maintained close to the incubation temperature treatments, and in a period of 3–4 minutes after removal from the incubators.

### Plasma hormones

After obtaining body surface and rectal temperatures, blood samples of the 24 chicks per treatment were collected by branchial vein puncture (approximately 1.5 ml of blood) and stored in Eppendorf tubes, containing EDTA. Blood was centrifuged at 2000 rpm for 10 min at 4°C to obtain plasma. Plasma was stored at -70°C until analysis. Plasma samples were analyzed for growth hormone (GH, ng/ml), 3,5,3'-triiodo-l-thyronine (T_3_, ng/ml) and 3,5,3',5'-tetraiodo-l-thyronine (T_4_, ng/ml). Total concentrations of T3, T4 and GH were measured by ELISA method using the T3 AccuBind ELISA KIT (Catalog # 125-300B, Monobind, Lake Forest, CA); the T4 AccuBind ELISA KIT (Monobind, Catalog # 225-300B); and a chicken growth hormone ELISA kit (Catalog # MBS266317, MyBioSource, San Diego, CA), respectively and a gamma radiation counter (Gamma-C12, Diagnostic Products Corp., Los Angeles, CA).

### Yolk free body mass (YFBM) and skin and feather analysis

Immediately after blood collection, chicks were weighed to obtain body mass, after which residual yolk was removed and hatchling yolk free body mass (YFBM) was obtained. Skin samples were removed using scissors and forceps from the neck (10 x 15 mm), back (between the insertion point of the wings) and breast (20 x 25 mm), and external region of the thigh (10 x 15 mm). All skin samples were immediately fixed in Bouin’s solution for 24 hour at room temperature, washed in distillated water and processed according to routine methods for light microscopy. Briefly, skin samples were dehydrated in a series of increasing ethanol concentrations (70%, 80%, 90%, 95% and 100%), diaphanized in ethanol-xylene solution (1:1) followed by xylene (100%, 3x), and embedded in a mixture of xylene-paraffin (1:1) followed by paraffin, spending approximately 45 minutes in each solution. Semi-serial 6-μm-thick cross-sections were prepared and stained with Masson`s Trichrome for morphological analysis (five cross-sections/slice). Measurements of epidermis, dermis and total skin thickness as well as blood vessel perimeter and number of blood vessels per area (area: 50.700 μm^2^) in the dermis were made using images obtained and examined using an image capture and analysis system (Leica QWin; Leica Imaging Systems, Wetzlar, Germany). Thirty measurements of each variable were considered per body region per hatchling in each slice. Feather weight was determined after manual removal of all down feathers from the body. Feather weight was calculated as the difference between body mass with and without feathers. Feather mass was expressed in grams and as a percentage of YFBM. Additionally, feather samples were excised from the back, breast and dorsal region of the neck (between the insertion point of the wings) and utilized for obtaining barbule length and barb number, length and width, using the Leica QWin image capture and analysis system. A total of 15 feathers per body region per hatchling and 10 barbs and 10 barbules per feather were measured.

### Thermal preference test

In one and two-day old male chicks a thermal preference test was executed, based on the methodology used previously by other authors [24, 32]. Two test chambers with similar dimensions (length x width x height: 160 x 60 x 50 cm), a thermal gradient system, temperature monitoring and bird location monitoring were used. Each test chamber consisted of a thin wall of aluminum, a thicker wall of medium-density fiberboard (MDF), a roof of transparent acrylic, and a grating floor of aluminum over an aluminum tray. Two electrical resistances (1000 W each) were fixed in the floor, giving rise to a thermal gradient from 19°C to 40°C along the chamber length. Air temperature and bird position in the test chamber were registered by twelve thermal sensors and twelve infrared sensors, respectively, along the chamber’s length. Data on the air temperature of the chamber and bird position were obtained and stored each minute during the measuring period. Both test chambers were used simultaneously. During each test, two chicks from the same treatments and age were put together into each test chamber. A preliminary study had shown that using one chick per test resulted in immobility of the chicks and absence of exploration of the thermal gradient within the chambers. When two chicks were used at the same time, such immobility did not occur, and both chicks explored the temperature gradient remaining close to each other most of the time. In addition, the preliminary study showed that chick position in the middle of the chamber had no effect on chick dislocation within the chamber. Thus, during the first 30 minutes of the test, chicks were positioned in the middle of the chamber and had the opportunity to explore it. After completion of this first period, the chicks were repositioned in the central region of the chamber and a new period of 60 minutes followed, during which temperature and chick position within the chamber were recorded. After the tests, a preferred ambient temperature was established for each chick, based on the air temperature of the chamber in which the bird remained for the longest time. In case the chick remained for a similar amount of time at two or more temperatures, the average ambient temperature was calculated. Before the thermal preference test, one day chicks were maintained in hatchers (Premium Ecológica, BH, MG, Brazil) at the same temperature as the incubation temperature treatment they originated from. Two-days old chicks remained in chick brooders, with electric heating done by infrared lamps (40W) (Premium Ecológica 1200, BH, MG, Brazil), receiving water and feed (based on corn and soybean meal, ME: 2,883Kcal/kg and BP: 21.27%; [33]) *ad libitum*. Because no significant differences in the preferred temperatures were found between the one and two days old chicks in all treatments, chicks of both ages were analyzed as one group (LT, CK, HT 1 and 2 days old chicks: 31.26±0.62 and 31.12±0.48°C, P = 0.3678; 31.81±0.85 and 31.5±1.64°C, P = 0.2032; 32.87±0.77 and 32.62±0.84°C, P = 0.4548; respectively).

### Statistical analyses

All measured variables were subjected to statistical analysis using the SAS 9.2 software package [34]. Model assumptions were proven for both means and residuals. Hatchability was tested with a logistic procedure with incubation temperature (Treatment) as a factor. Measured temperatures (air incubator, eggshell, skin surface, rectal) and skin, feather and hormonal characteristics were subjected to the GLM procedure for analysis of variance using Treatment as a fixed factor. Means were compared after correction with Tukey’s test for multiple comparisons. Results are reported as means ± SD and the means were considered different at P < 0.05. For all variables the individual egg or chick was considered as the experimental unit. Only male chickens were used, whereas female chickens were removed from the experiment.

## Results

### Induced changes in eggshell temperature during fetal development

**Fig 1:**
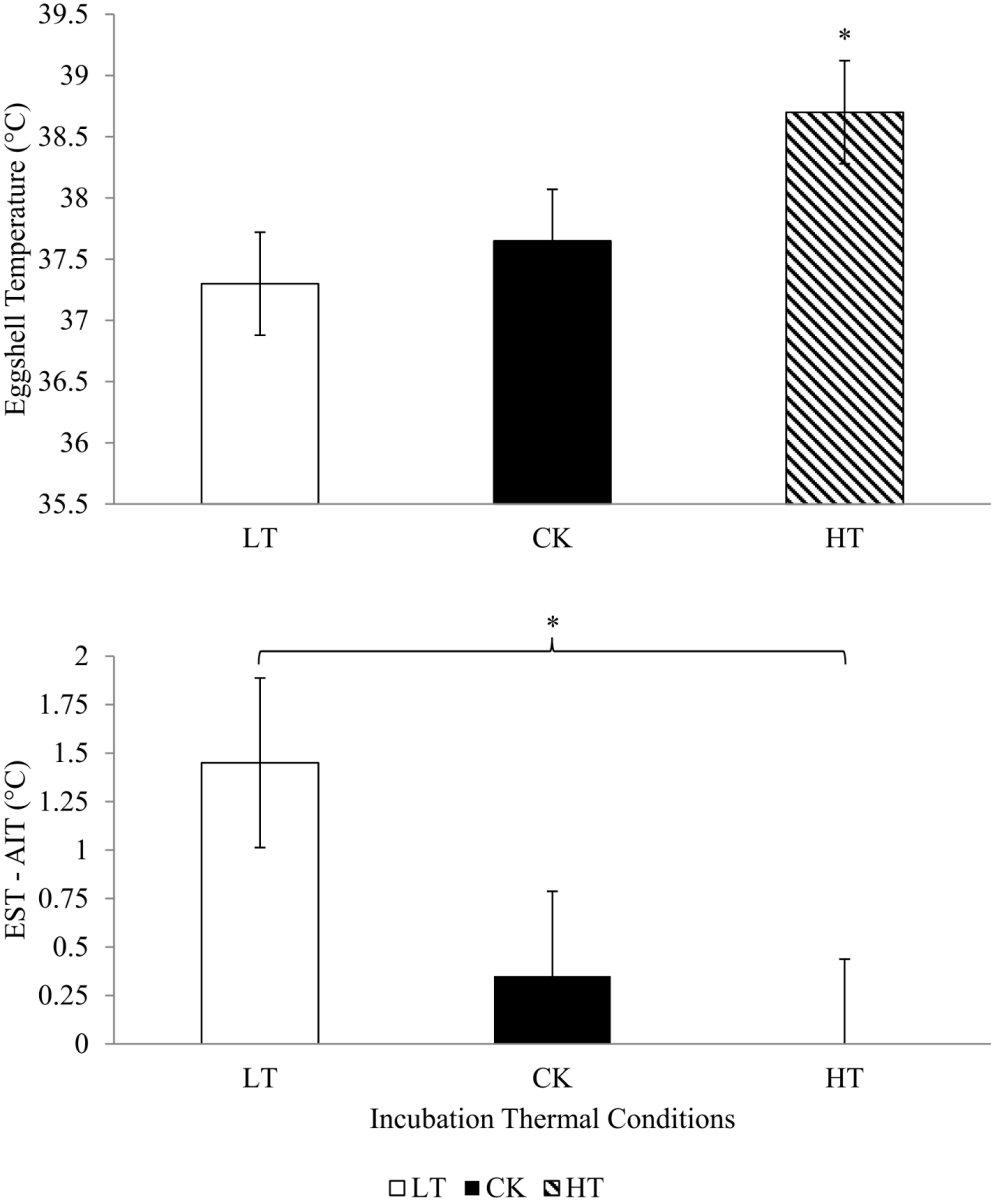
A) Average eggshell temperature from day 13 to hatching in the LT (36°C), CK (37.5°C) and HT (39°C) treatments. B) Mean differences between eggshell and incubator air temperature from day 13 to 21 of incubation in the LT (36°C), CK (37.5°C) and HT (39°C) treatments. *Indicates a difference between means (*P* < 0.05); reported values represent the means ± SD (n = 10 eggs/treatment).

Incubation temperature treatments (LT: 36°C; CK: 37.5°C; HT: 39°C) were applied from day 13 to day 21 of incubation. During this period, mean eggshell temperature of CK and LT eggs did not differ significantly, but both had significantly lower eggshell temperature (P < 0.05) than HT eggs (Fig 1A). In the LT treatment, the mean difference between eggshell and incubator air temperature was 1.30°C, whereas this difference was zero for HT eggs (P < 0.05). The difference between eggshell and incubator air temperature of the control group was intermediate between that of both other groups (0.4°C) and was not significantly different from them (Fig 1B).

### Incubation duration, hatchability and hatchling body mass

No significant effect of incubation treatment could be observed on hatchling body mass (LT = 48.50 g, CK = 48.94 g, HT = 48.98 g, P = 0.81, CV = 6.58%). However, YFBM was lower for HT chicks compared to LT chicks, while CT chicks had no significant difference in YFBM compared to these two treatments (LT = 40.62 g, CK = 40.15 g, HT = 39.57 g, P = 0.0008, CV = 15.09%). Yolk-sac mass was higher for HT than LT chicks and both did not differ from yolk sac mass of CT chicks (LT: 7.85 g, CT: 8.81 g, HT: 9.41, P = 0.0008, CV = 5.4%). Duration of incubation was significantly longer for the LT treatment compared to the CT and HT treatments, which had similar durations (LT = 520 h, CK = 509 h, HT = 503 h, P < 0.0001, CV = 2.94%). Hatchability in the cold and control treatments was similar, but lower than in the heat treatment (LT = 67.6%, CK = 68.1%, HT = 75.7%, P < 0.001).

### Body surface and rectal temperatures

Head (P = 0.002) and back surface (P = 0.03) temperatures increased with incubation temperature (Table 1), whereas neck surface temperatures were significantly higher in HT treatment compared to the LT and CK treatments, which did not differ from each other. Rectal temperature and thigh and breast skin temperature were not affected by incubation treatment (P > 0.05). Comparison among body regions showed that in all incubation temperature treatments, body surface temperatures were lower than the rectal temperature, whereas head temperature was higher than neck, back, thigh and breast surface temperatures, which did not differ (P < 0.0001 for LT, CK and HT).

**Table 1:** Body surface and rectal temperatures of hatchlings obtained from eggs exposed to low temperature (LT; 36°C), control temperature (CK, 37.5°C) or high temperature (HT, 39°C) treatments from day 13 to 21 of incubation (means ± SD).

Variables	Incubation Thermal Condition				
(N = 24 chicks/treatment)	LT	CK	HT	*P value*	CV(%)
Rectum	38.0±0.7	37.9±0.3	38.2±0.4	0.63	9.22
Head	32.1±0.9^c^	33.7±0.7^b^	34.3±1.0^a^	0.002	3.80
Neck	30.6±0.4^b^	31.5±0.5^a^	32.0±0.6^a^	0.04	3.94
Back	30.8±0.3^c^	31.9 ±0.4^b^	32.4±0.3^a^	0.03	4.90
Thigh	31.3±0.4	31.5±0.5	31.8±0.4	0.18	5.01
Breast	30.1±0.6	30.7 ±0.4	30.9±0.6	0.69	4.28

^a-c^ Values within a row lacking a common superscript differ (*P* < 0.05).

### Feather weight and morphology

Incubation treatment had no effect on absolute or relative feather weight, nor on barbule length or barb number, length or width (Table 2).

**Table 2:** Feathering characteristics of hatchlings obtained from eggs exposed to low (LT, 36°C), control (CK, 37.5°C) or high (HT, 39°C) temperature treatments from day 13 to 21 of incubation (means ± SD).

Variables	Incubation Thermal Condition				
(N = 24 chicks/treatment)	LT	CK	HT	*P value*	CV(%)
Weight					
(g)	1.94±0.10	1.70±0.08	1.58±0.11	0.44	39.36
(%)*	3.72±0.12	3.24±0.23	2.98±0.17	0.43	39.45
(%)**	3.88±0.21	3.70±0.14	3.47±0.26	0.52	22.27
Neck					
Barb number	11.80±1.02	12.89±1.89	12.29±1.71	0.11	2.29
Barb length (mm)	15.69±1.03	14.94±1.34	14.27±1.51	0.48	8.94
Barb width (mm)	1.53±0.17	1.56±0.11	1.61±0.19	0.81	9.33
Barbule length (mm)	1.19±0.15	1.17±0.14	1.13±0.11	0.42	4.21
Back					
Barb number	13.97±1.09	14.08±1.17	14.05±1.22	0.32	6.55
Barb length (mm)	14.06±0.99	13.89±1.04	13.95±1.01	0.36	8.67
Barb width (mm)	1.76±0.19	1.71±0.21	1.70±0.25	0.12	8.3
Barbule length (mm)	1.53±0.14	1.48±0.17	1.50±0.17	0.38	18.69
Breast					
Barb number	15.97±1.02	14.67±1.15	15.61±0.98	0.25	5.65
Barb length (mm)	12.23±1.09	11.05±1.23	11.94±1.02	0.44	9.25
Barb width (mm)	2.00±0.16	1.82±0.13	1.80±0.13	0.20	7.0
Barbule length (mm)	1.83±0.34	1.38±0.19	1.38±0.21	0.24	21.70

* calculated in relation to pre-incubation egg mass;

** calculated in relation to yolk-free body mass at hatch.

### Skin thickness and vascularity

**Fig 2:**
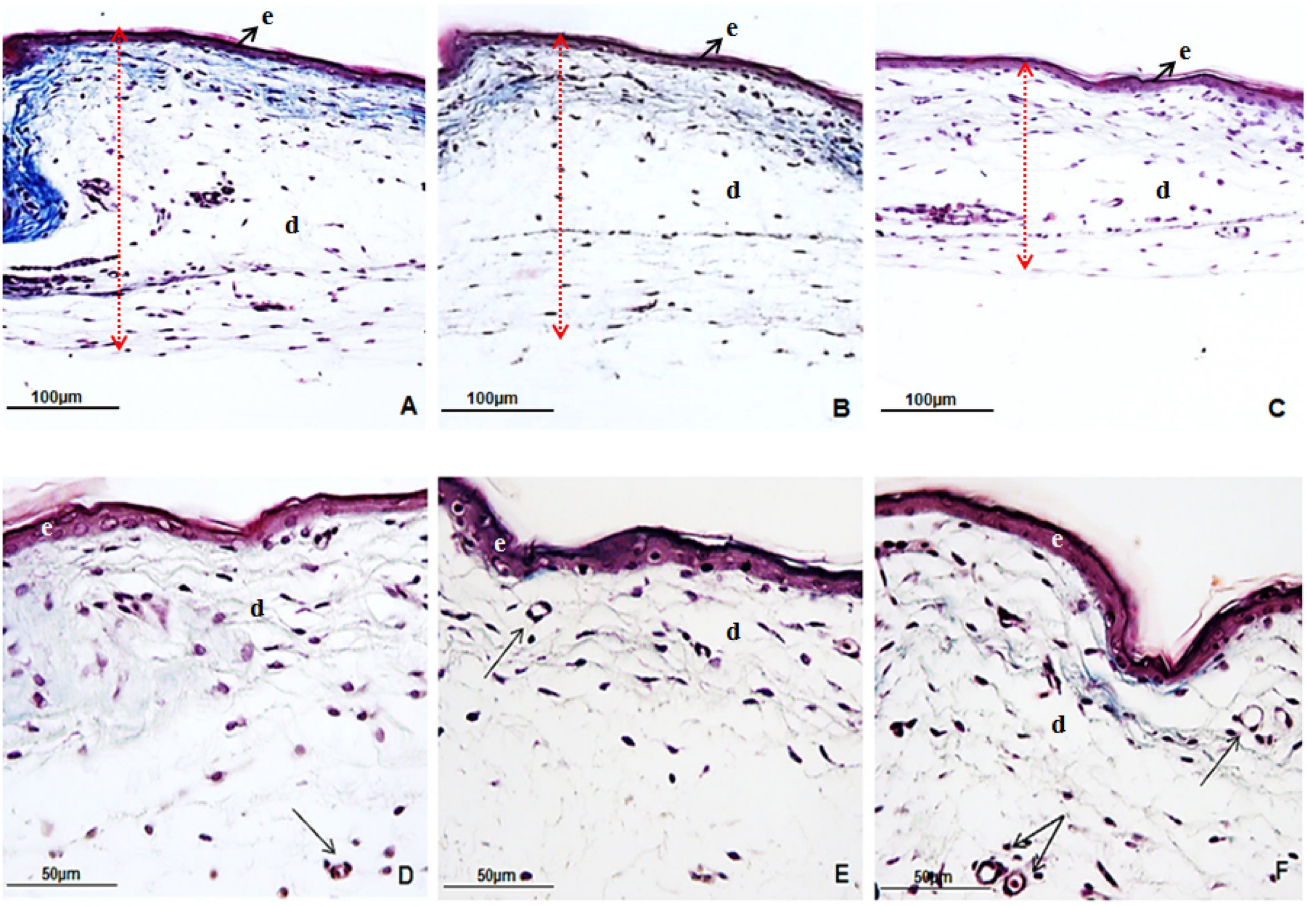
Skin histological sections of broiler hatchlings submitted to low (LT, 36°C, A and D), control (CK, 37.5°C, B and E) or heat (HT, 39°C, C and F) incubation treatments during fetal development. A-C) Neck skin thickness was lower in the heat (C) than in cold and control treatments (A and B, respectively). D-F) Blood vessels perimeter increased with incubation temperature. e: epidermis. d: dermis. Black arrows indicate cross sections of blood vessels. Red double arrows indicate skin thickness.

HT treatment resulted in lower skin and dermis thicknesses in all analyzed body regions, compared to the LT and CK treatments, which did not differ from each other (Table 3, Fig 2A–2C). Incubation treatment had no significant effect on epidermis thickness.

**Table 3:** Skin, epidermis and dermis thickness (μm) and blood vessel number (counts per field) and perimeter (μm) of chicks obtained from eggs exposed to low (LT, 36°C), control (CK, 37.5°C) or high (HT, 39°C) temperature treatments from day 13 to 21 of incubation (means ± SD).

Variables	Incubation Thermal Conditions				
(N = 24 chicks/treatment)	LT	CK	HT	*P value*	CV (%)
Neck					
Skin	432.81±37.14^a^	469.82±43.25^a^	360.85±39.21^b^	0.004	8.48
Epidermis	14.49±1.75	20.40±1.81	16.48±2.45	0.27	6.02
Dermis	418.34±43.28^a^	449.43±35.61^a^	343.72±59.14^b^	0.004	8.59
Blood Vessel Number	1.67±0.24^b^	2.00±0.19^a^	2.33±0.31^a^	0.04	17.66
Blood Vessel Perimeter	47.39±6.41^b^	54.88±8.56^a^	55.42±6.18^a^	0.05	9.52
Back					
Skin	479.38±31.99^a^	443.81±42.14^a^	370.54±33.17^b^	0.006	4.98
Epidermis	12.31±1.93	16.38±1.01	16.67±1.89	0.18	10.84
Dermis	462.79±30.88^a^	427.50 ±34.17^a^	353.80±37.77^b^	0.004	5.25
Blood Vessel Number	1.98±0.20	2.14±0.17	2.26±0.16	0.07	8.23
Blood Vessel Perimeter	53.15±6.14^b^	64.71±5.67^a^	69.15±6.14^a^	0.05	14.46
Breast					
Skin	563.01±39.17^a^	535.22±31.78^a^	474.52±36.56^b^	0.005	8.51
Epidermis	20.92±2.01	17.68±1.87	15.88±1.91	0.08	4.11
Dermis	542.09±41.13^a^	527.66±37.69^a^	458.53±35.23^b^	0.002	9.17
Blood Vessel Number	1.45±0.16	1.59±0.13	1.62±0.15	0.09	15.09
Blood Vessel Perimeter	51.10±9.17^b^	56.98±8.33^b^	65.57±8.04^a^	0.03	9.05
Thigh					
Skin	387.29±45.55^a^	392.46±41.17^a^	338.75±39.38^b^	0.009	4.57
Epidermis	18.26±1.88	17.32±1.94	16.51±1.99	0.55	2.52
Dermis	368.38±35.67^a^	375.29±42.19^a^	323.32±43.11^b^	0.02	4.86
Blood Vessel Number	1.15±0.23	1.47±0.17	1.62±0.18	0.14	18.90
Blood Vessel Perimeter	44.11±8.76^c^	54.48±7.14^b^	69.80±7.78^a^	<0.001	3.70

^a-c^ Values within a row lacking a common superscript differ (*P* ≤ 0.05).

Blood vessel number and perimeter in the dermis were evaluated as a measure of skin vascularity in the neck, back, breast and thigh (Table 3). Number of blood vessels in the neck area of hatchlings was lower (P = 0.04) in the LT treatment in comparison to the CK and HT treatments, which did not differ from each other. In all other regions, number of blood vessels did not differ significantly among the incubation treatments. Incubation treatment had a significant influence on blood vessel perimeter (P ≤ 0.05) in all analyzed body regions, but effects differed per region (Table 3). Neck and back blood vessel perimeters in the dermis were smaller in LT chicks compared to the CK and HT chicks. In the breast dermis, blood vessel perimeters were smaller for the LT and CK chicks compared to the HT hatchlings (P = 0.03), whereas for the thigh dermis the perimeter of blood vessels increased with incubation temperature (Fig 2D–2F).

### Plasma hormones

**Fig 3:**
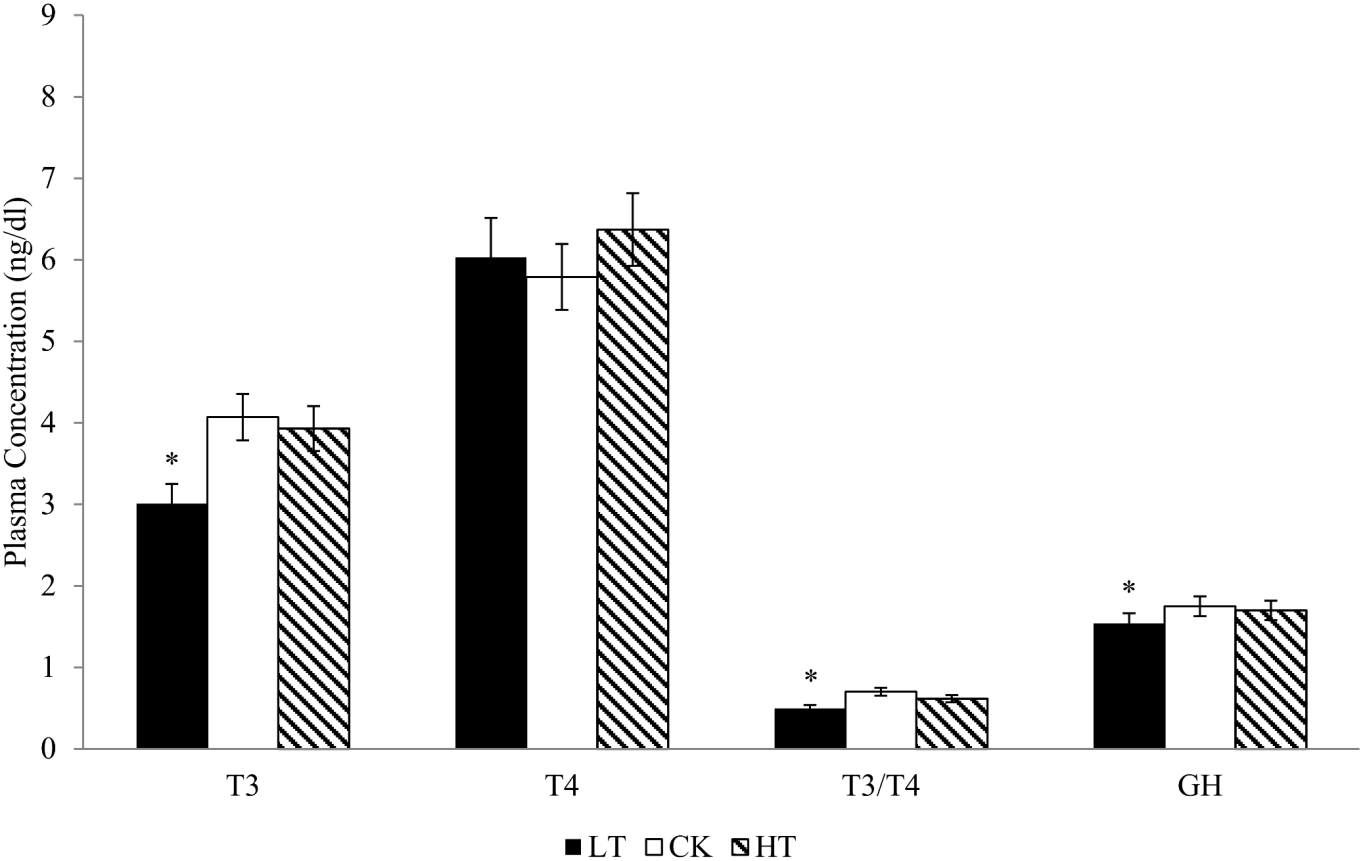
Plasma concentrations of triiodothyronine (T_3_, ng/ml), thyroxine (T_4_, ng/ml), T3/T4 ratio and growth hormone (GH, ng/ml) of hatchlings obtained from eggs exposed to low (LT, 36°C), control (CK, 37.5°C) or high (HT, 39°C) temperature treatments from day 13 to 21 of incubation. * Indicates a difference between means (*P* < 0.05); values represent the means ± SD (n = 24 chicks/treatment).

Plasma T3, T3/T4 ratio and GH levels were significantly lower (P < 0.05) in LT chicks compared to the CK and HT chicks, which did not differ from each other (Fig 3). Plasma T4 levels did not differ among treatments.

### Preferred ambient temperature

**Fig 4:**
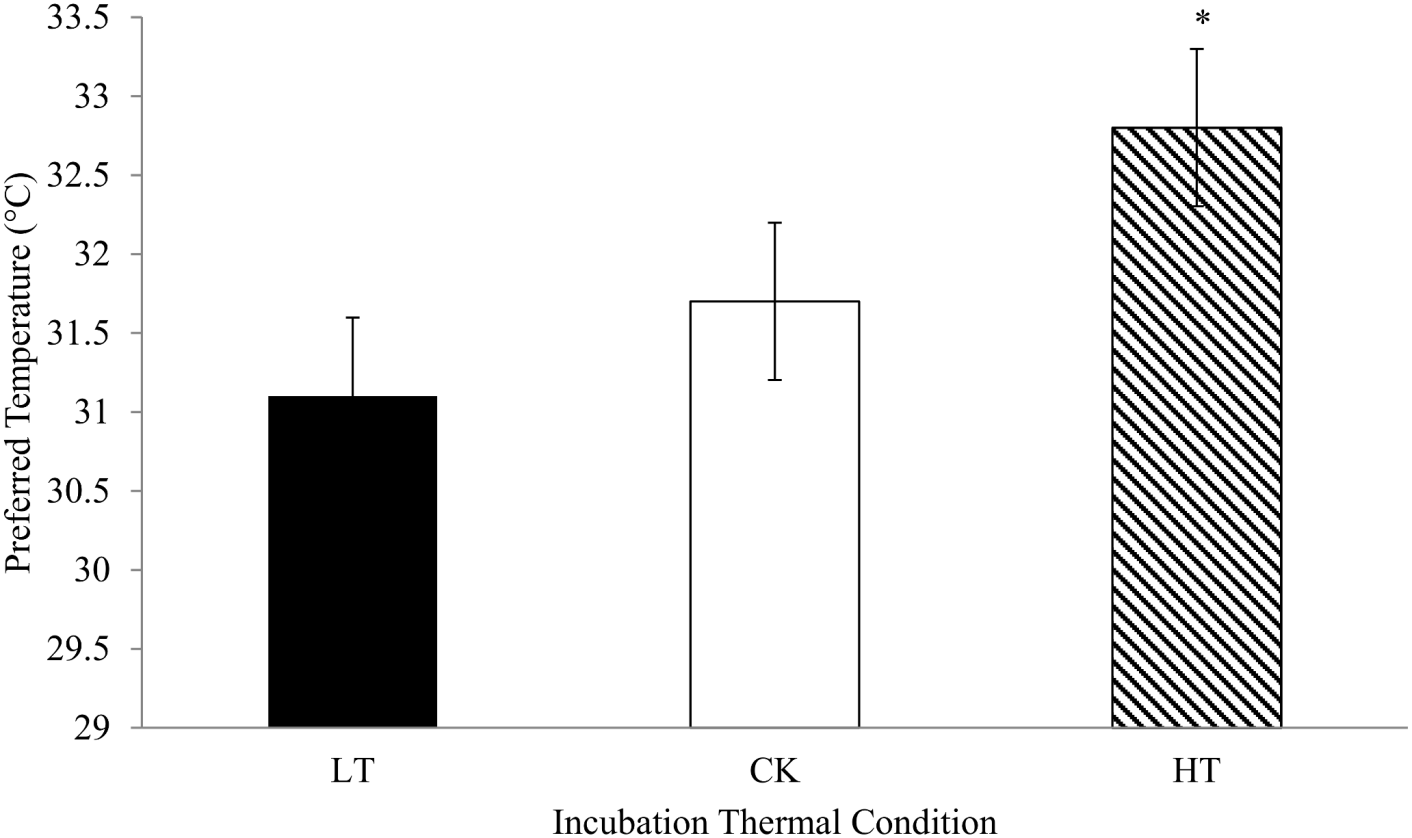
Preferred ambient temperature of hatchlings submitted to low (LT, 36°C), control (CK, 37.5°C) or high (HT,39°C) temperature treatments from day 13 to 21 of incubation. * Indicates a difference between means (*P* < 0.05); values represent the means ± SD (n = 16 chicks/treatment).

HT chicks preferred a higher ambient temperature (P < 0.05) compared to chicks from the two other incubation treatments, which did not differ from each other (Fig 4).

## Discussion

### Effects of increased incubation temperature

The present study analyzed whether exposure to increased or decreased temperature throughout the second half of incubation (day 13 to hatching) can induce morphophysiological alterations, which, in turn, affect chick thermal preference. Compared to the control treatment (CK, 37.5°C), the high temperature treatment (HT, 39.0°C) resulted in increased hatchability, hatchling head and breast surface temperatures, and breast and thigh dermal vascularization, while it also resulted in decreased skin thickness in all analyzed body regions. Additionally, the HT treatment increased chick’s preferred ambient temperature by approximately 1°C. No differences were observed in rectal temperature, body weight, feather morphology and plasma hormone concentrations (T3, T4, and GH) between the HT and CK treatments.

Regarding the HT treatment, the current work differs from previous ones in two main points. First, the current study demonstrated an increase in the hatchability of HT eggs, without alteration in hormone concentrations between the HT and CK treatments, whereas other studies have reported decreased hatchability [1, 15, 16, 22, 35] and lower T3 plasma concentrations after high incubation temperatures [16, 22, 36]. These ambiguous results might be explained by a lack of difference between incubator air and eggshell temperatures in our study. Incubating eggs at an incubator air temperature matching eggshell temperature, as occurred throughout our HT treatment, improved hatchability without altering plasma hormone rates and incubation period. Second, although the bird skin has considerable potential to respond to environmental stimuli in order to regulate heat maintenance or dissipation [5, 6, 10, 37, 38], no studies have analyzed changes in poultry skin thickness and vascularization in relation to incubation temperature. To lose heat, birds elevate the wing to expose its sparsely feathered undersurface [39] to increase the blood flow to the skin, as a result of decreased vascular resistance [5, 40]. Furthermore, heat-acclimated pigeons presented increased epidermis thickness and dermis vascularization in the back and abdomen [10]. Other more recent studies analyzed core and surface temperature changes and attribute surface temperature changes to vasodilatation or vasoconstriction in distinct parts of the bird body, including the feet [38, 41, 42], but without measurements on skin characteristics. However, all above mentioned studies analyzed thermal response of birds after hatch and the most of them did not investigated changes in the morphological structure of the skin. Thus, alterations observed in the current study in the broiler chick skin are association with a higher preferred ambient temperature in response to incubation temperature manipulation constitute a novel finding. It is known body regulates the conductive heat exchange with the environment by controlling the cutaneous blood flow, which determines the skin temperature, and the insulating blanket property of the skin depends on its thickness and vascularity [43]. From this view, the thinner and more vascularized integument observed in the current study after HT incubation apparently represents an adaptive embryonic response that would drive greater heat loss. This idea is supported by the increase in surface body temperature in the head and back of chicks, without a change in internal body temperature. The distinct thermal sensitivity of body regions [4, 6, 7, 38] and distinct skin thickness and vascularization may explain the increased surface temperature of the head and back and not the neck, thigh and breast area.

Thyroid hormones have significant influence on the metabolism, development and thermoregulatory mechanisms of the chicks in the post-hatch period [26] and consequently on metabolic heat production. By Van ´t Hoff law, chicks of heat treatment would have increased metabolism and, consequently shorter incubation period [44]. However, in the present study, chicks of the heat treatment from day 13 of incubation onward did not show differences in the plasma T3 and T4 levels at hatching and in the growth hormone rate and rectal temperature compared to chicks of the control treatment. The body uses heat exchange mechanisms as well as the metabolic rate of energy production to ensure the maintenance (43). The similar hormone rates suggest similar metabolic and growth rates and, consequently similar heat production, and this appear to be reinforced by similar incubation length and body mass showed in the present and also in a previous study [45]. In our view, the thinner and more vascularized skin occurrence without thyroid hormone changes allowed HT chicks to major conductive heat loss, indicated by the higher temperature at the body surface, enabling maintenance of body temperature without regulatory metabolic changes. On the other hand, a thinner and more vascularized integument, leading to greater heat loss, may also have driven the choice for a higher ambient temperature, as a behavioral adjustment that helps hatchlings maintain homeothermy, which is in agreement with an earlier study [23]. If these changes have long lasting effects along the age of the chicks, thermal manipulation from the 13th day of incubation may be a tool to increase the thermotolerance of chicks in environments where the shed temperature control is one of the obstacles to poultry production. The long-lasting effects of high incubation temperature on skin characteristics and thermal preference may help chicks to maintain body temperatures at normal or at least not lethal levels, even during periods of high ambient temperature in later life, as shown before [10] Previous studies have showed a post-hatch circadian rhythm of thyroid hormones in broilers at thermoneutral and heat conditions [46, 47]. Here, thyroid hormone rates were analyzed at hatch only. Thus, it is important to consider that the plasma T_3_, T_4_ and T_3_/T_4_ levels reported in the present study could not completely discern the impact of heat incubation temperature.

### Effects of decreased incubation temperature

In contrast with the results of the HT treatment discussed above, the low temperature incubation treatment (LT, 36°C) resulted in reduced head, neck and back surface temperatures, reduced neck, back and thigh dermal vascularization, decreased T3 and GH plasma concentrations and longer incubation duration compared to the CK treatment. The LT treatment did not have an effect on body weight at hatch or on preferred ambient temperature in day-old chickens compared to the CK treatment. These findings differ from those obtained by other authors [25]**,** which showed that broiler chicks obtained from eggs incubated at low temperature (34.5°C) during day18 to 20 of incubation preferred an 1°C ambient temperature higher than chicks obtained from eggs incubated at an usual control temperature (37.5°C). At the same time, current results are supported by results of previous work on low incubation temperature that showed that ducklings require longer incubation times and that broiler chicks exhibit lower T3 levels and T3/T4 ratios [48]. Another study [22], however, reported no change in hormone concentrations of broiler chicks that had been subjected to intermittent periods of low temperature during embryonic development. These previous results cannot be directly compared to ours, as the period, frequency and intensity of temperature alteration were different. The reduction we observed in plasma T3 and in the T3/T4 ratio after LT treatment might be the result of a decrease in liver deiodinase activity, which, in turn, impairs the conversion of T4 to T3 and increases incubation time [49].

Thyroid hormone levels are considered good indicators of an animal’s metabolic state [50], whereas GH levels are an indicator of growth rate. We observed that reductions in T3 and GH occurred along with longer incubation duration in the LT treatment. These findings indicate that, at lower temperatures, metabolism and growth rates slow down, delaying embryonic development. Moreover, we found that, in contrast with the HT treatment, the LT treatment resulted in chicks with lower neck, back and thigh dermal vascularity. Considering that heat exchange occurs from bodies at a higher temperature to a lower-temperature environment [51], the larger difference between incubator air and eggshell temperatures in the LT treatment resulted in increased heat loss from eggs. Increased heat loss as well as lower metabolic rates may have, in turn, elicited physiological adjustments, such as the reduction in vascularization that was observed. Chick surface temperature was indeed lower in LT chicks, compared to CK chicks, in areas with lower vascularization, indicating a reduced potential for heat exchange. Although LT chicks preferred an ambient temperature equal to that chosen by control birds, if these changes in the skin remain along the age of the chickens, it is possible that such changes are an adaptive adjustment that improves their post-hatch survival, especially in cold regions. However, to confirm this hypothesis, studies are needed to analyze these parameters along the rearing.

Altogether our results are consistent with the hypothesis that incubation temperature is an important factor influencing hormone levels and morphophysiological characteristics associated with chick behavioral adjustments. Our findings demonstrate, for the first time, that precocial birds can react with adaptive adjustments to long and continuous period (from day 13) of higher and lower incubation temperatures than normal.
